# Supplementary material for: How do users of a ‘digital-only’ contraceptive service provide biometric measurements and what does this teach us about safe and effective online care? A qualitative interview study
Source: BMJ Open. 2020 Sep 29;10(9):e037851. doi: 10.1136/bmjopen-2020-037851 (PMC7526275; doi:10.1136/bmjopen-2020-037851)
Supplement: Supplementary data [file bmjopen-2020-037851supp001.pdf]

Appendix A – Information provided by the service on the risks of taking the COCP with high blood pressure

SH:24

Sexual health Contraception Advice & support

Order combined contraceptive pill

1 Start your order
2 About you
3 Your health
4 Your health history
5 Contact & delivery details
6 More about you
7 Complete your order

Are you able to provide your blood pressure? i

Yes No

What is your blood pressure? i

Your health may be at risk if you provide inaccurate answers - if you are unsure, please talk to one of our clinicians by text (07860 041 233).

Top number (Systolic) systolic

Bottom number (Diastolic) diastolic

A reading of 140/90 or above is known as high blood pressure.

Women aged 30-34 who have high blood pressure and take the combined pill:

- are more than 5 times more likely to have a stroke
- are 6 times more likely to have a heart attack
- double the risk of heart attack or stroke every time their blood pressure increases by 20/10

A clinician will call you to discuss your options if your blood pressure is high.
