# Supplementary material for: How do users of a ‘digital-only’ contraceptive service provide biometric measurements and what does this teach us about safe and effective online care? A qualitative interview study
Source: BMJ Open. 2020 Sep 29;10(9):e037851. doi: 10.1136/bmjopen-2020-037851 (PMC7526275; doi:10.1136/bmjopen-2020-037851)
Supplement: Supplementary data [file bmjopen-2020-037851supp002.pdf]

Appendix B: Qualitative paper coding tree

Theme	Sub-themes	Notes
The practical realities of obtaining contraception from traditional services and the choice to order the pill online	An alternative to inconvenient existing options<br><br>Fits into my lifestyle<br><br>A solution to an urgent situation	Navigating access to contraception with time pressure - Time and urgency is of fundamental importance in this assessment. Reference negative case study of person who did not have time pressure
A new contraceptive service option generates new work, new roles and new responsibilities.	Social work (Drawing on existing relationships to access a BP reading)<br><br>Ethical work (Negotiating an ethical position under pressure to obtain contraception)<br><br>Research work (online research to find out what is normal and the consequences of taking the pill with high BP).	By changing the frame of access and the nature of the interface with the health system we have created a new option with a new set of practical realities.<br><br>No existing pathways, so created new pathways, used chance events. Translating past experience into this new context.<br><br>Differences between strategies to measure BP (unfamiliar) and height/weight (familiar)
Ideas about blood pressure and its measurement.	Blood pressure as an intangible concept<br><br>Blood pressure as linked to own health and normality – from past clinical experiences<br><br>BP as a medical thing whereas BMI is more in the public domain.<br><br>Blood pressure and how recent it needs to be?	Consequences of these ideas for choice of measurement strategy.
Responsibility and risk	Negotiating a clinical relationship with a faceless service<br><br>The characteristics of the digital interface that supported or did not support effective communication.	
