# Supplementary material for: How do users of a ‘digital-only’ contraceptive service provide biometric measurements and what does this teach us about safe and effective online care? A qualitative interview study
Source: BMJ Open. 2020 Sep 29;10(9):e037851. doi: 10.1136/bmjopen-2020-037851 (PMC7526275; doi:10.1136/bmjopen-2020-037851)
Supplement: Author's manuscript [file bmjopen-2020-037851.draft_revisions.pdf]

BMJ Open is committed to open peer review. As part of this commitment we make the peer review history of every article we publish publicly available.

When an article is published we post the peer reviewers' comments and the authors' responses online. We also post the versions of the paper that were used during peer review. These are the versions that the peer review comments apply to.

The versions of the paper that follow are the versions that were submitted during the peer review process. They are not the versions of record or the final published versions. They should not be cited or distributed as the published version of this manuscript.

BMJ Open is an open access journal and the full, final, typeset and author-corrected version of record of the manuscript is available on our site with no access controls, subscription charges or pay-per-view fees (http://bmjopen.bmj.com).

If you have any questions on BMJ Open's open peer review process please email info.bmjopen@bmj.com

BMJ Open

How do users of a 'digital-only' contraceptive service provide biometric measurements and what does this teach us about safe and effective online care? A qualitative interview study

Journal:	*BMJ Open*
Manuscript ID	bmjopen-2020-037851
Article Type:	Original research
Date Submitted by the Author:	18-Feb-2020

Complete List of Authors:	Baraitser, Paula; SH:24<br>McCulloch, Hannah; King's College London, King's Centre for Global Health and Health Partnerships<br>Morelli, Alessandra; King's College London, King's Centre for Global Health and Health Partnerships<br>Free, Caroline; London School of Hygiene and Tropical Medicine, Public Health interventions Unit

Keywords:	Telemedicine < BIOTECHNOLOGY & BIOINFORMATICS, Quality in health care < HEALTH SERVICES ADMINISTRATION & MANAGEMENT, SEXUAL MEDICINE

SCHOLARONE™
Manuscripts

I, the Submitting Author has the right to grant and does grant on behalf of all authors of the Work (as defined in the below author licence), an exclusive licence and/or a non-exclusive licence for contributions from authors who are: i) UK Crown employees; ii) where BMJ has agreed a CC-BY licence shall apply, and/or iii) in accordance with the terms applicable for US Federal Government officers or employees acting as part of their official duties; on a worldwide, perpetual, irrevocable, royalty-free basis to BMJ Publishing Group Ltd ("BMJ") its licensees and where the relevant Journal is co-owned by BMJ to the co-owners of the Journal, to publish the Work in this journal and any other BMJ products and to exploit all rights, as set out in our licence.

The Submitting Author accepts and understands that any supply made under these terms is made by BMJ to the Submitting Author unless you are acting as an employee on behalf of your employer or a postgraduate student of an affiliated institution which is paying any applicable article publishing charge ("APC") for Open Access articles. Where the Submitting Author wishes to make the Work available on an Open Access basis (and intends to pay the relevant APC), the terms of reuse of such Open Access shall be governed by a Creative Commons licence – details of these licences and which Creative Commons licence will apply to this Work are set out in our licence referred to above.

Other than as permitted in any relevant BMJ Author's Self Archiving Policies, I confirm this Work has not been accepted for publication elsewhere, is not being considered for publication elsewhere and does not duplicate material already published. I confirm all authors consent to publication of this Work and authorise the granting of this licence.

For peer review only

How do users of a 'digital-only' contraceptive service provide biometric measurements and what does this teach us about safe and effective online care? A qualitative interview study

Paula Baraitser, Hannah McCulloch, Alessandra Morelli, Caroline Free

Paula Baraitser, Paula_baraitser@mac.com (corresponding author) Paula Baraitser, SH:24 35A Westminster Bridge Road, London, SE1 7JB, +442076202250

Hannah MCulloch, Kings Centre for Global Health and Health Partnerships, King's College London, London, UK hannah.mcculloch@kcl.ac.uk

Alessaandra Morelli, King's Centre for Global Health and Health Parterships, King's College London, London, UK King's College London, alessandra.morelli@kcl.ac.uk

Caroline Free, Department of Population Health, London School of Hygiene and Tropical Medicine, London, UK Caroline.Free@lshtm.ac.uk

Word count: 3090

Key words: digital health, sexual health, contraception, blood pressure

ABSTRACT

Objectives

- To describe user experience of providing biometric measurements to a 'digital only' contraceptive service to obtain a prescription for the combined oral contraceptive pill.
- To analyse this experience to generate recommendations for the design of 'digital only' online contraceptive services to maximise clinical safety and user acceptability.

Setting

An online contraceptive service available free of charge to women in South East London, UK

Participants

20 participants who had ordered the COCP online. Our purposive sampling strategy ensured that we included participants from a wide range of ages and those who were and were not prescribed the COCP.

Intervention

A 'digital only' contraceptive service that prescribes the combined oral contraceptive pill after an online medical history and self-reported height, weight and blood pressure with pills prescribed by a GMC registered doctor, dispensed by an online pharmacy and posted to the user.

Design

For peer review only

Semi-structured interviews with a purposive sample of 20 participants who were already enrolled in a larger study of this service.

Analysis
Inductive, thematic analysis of the interviews assisted by NVivo qualitative analysis software.

Results
Users valued the convenience of 'digital-only care' but experienced measuring blood pressure but not height or weight as a significant barrier to service use. They actively engaged in work to understand and measure blood pressure through a combination of recent/past measurements, borrowed machines, health service visits and online research. They negotiated tensions around maintaining a trusting relationship with the service, meeting its demands for accurate information while also obtaining the contraception that they needed.

Conclusion
Digital strategies to build trusting clinical relationships despite a lack of face-to-face contact are needed in 'digital only' health services. This includes acknowledgement of work required, evidence of credible human support and a digital interface that communicates the health benefits of collaborating with an engaged clinical team.

STRENGTHS AND LIMITATIONS OF THIS STUDY

This is the first study that looks at user experience of use of online contraceptive services and is important because most UK women will use the contraceptive pill at some time in their lives and increasing numbers look to access this online.

It provides detailed accounts of women's work to measure their blood pressure prior to a prescription of the pill and to negotiate a relationship with a 'digital-only' service.

It offers clear recommendations for good practice within services that provide prescriptions without face-to-face consultations.
Like all qualitative research it offers in-depth accounts from small numbers of people and may not be representative of larger populations.

Background

People have always acted to maintain and improve their own health, but self-care within formal health systems is a recent development[1-2]. Early research and interventions on self-care within clinical systems focused on shared decision making and symptom management for those living with long term conditions[2-3]. More recently, complex self-care tasks, for example, blood sugar monitoring[4] or self-testing for sexually transmitted infections[5] are included. These tasks often happen outside clinical contexts with remote clinical support and require access to clinical technologies (such as blood sugar monitors or lancets to collect blood) and digital interfaces, that support technology use by storing data, providing information and facilitating remote communication between users and clinicians.

For peer review only

National and international health policy documents suggest that digitally enabled, self-care offers the potential to improve user experience with more convenient, more informative and more cost-effective services[6-9]. There is a growing body of literature on digital health services that support home self-monitoring of those with long term conditions such as diabetes or hypertension as an adjunct to health care from a face-to-face service[10-11] and on digital self-monitoring of biometric data (self-tracking) that occurs outside the health system. This literature suggests that outcomes of transferring clinical technologies to domestic spaces may be unpredictable as they are adapted by users for home settings, and framed within non-clinical understandings of health and health care[10-15]. There is less research on digitally facilitated self-care as a stand-alone intervention without a 'face-to-face' element[16]. This gap in the evidence has implications for the 'digital only' or 'digital first' services that are increasingly part of health policy[9].

Sexual health services have been early adopters of digitally enabled self-care[17], possibly because self-care is particularly attractive where stigma is a barrier to service access[1]. Online testing for sexually transmitted infection is now routine practice in many public health systems[5, 18, 19] and there is an expanding market for online contraception[20]. These services are 'online only' services where it is anticipated that the whole clinical pathway will be delivered online.

We studied a UK based, online sexual health service providing the combined oral contraceptive pill (COCP), delivered home by post after completion of a medical history and self-reporting of height, weight and blood pressure (BP). As an early example of 'digital only' facilitated self-care, we were interested in user experience of measuring and uploading BP, height and weight readings prior to online prescriptions. This is an example of 'pushed' self-tracking[10] or 'clinical self-tracking[11], where the service requires self-measurement as a condition of service delivery.

The measurement of BP and body mass index (BMI) prior to first prescription of COCP is recommended by national and international guidance[21-22]. Ethinyl oestradiol, (the oestrogenic component of most COCPs) stimulates an increase in hepatic protein production including angiotensinogen leading to a slightly reduced renal blood flow and therefore a slight increase in BP[23]. In about 2% of women BP may increase significantly after starting the COCP[24]. Since hypertension increases risk of cardiovascular disease, BP is monitored before first prescription of the pill and then annually and the COCP is contraindicated if BP is consistently above 140/90 mmHg. Obesity is similarly associated with risk of cardiovascular disease, and height and weight are also measured prior to COCP prescription. If BMI (weight in kilograms divided by height squared in metres) is equal to or greater than 35kg/m^2 then the risks of taking the pill are considered to outweigh the benefits[21]. In face-to-face clinical practice, biometric measurements are collected and recorded by health care professionals prior to prescribing, usually as a single measurement with repeat readings taken if the first reading is abnormal. In the online service users are asked to upload a recent reading.

Accuracy of biometric readings in this context is clinically important and may be influenced by user experience of this process[10]. We were therefore interested in how users experience the process of self-reported measurement and how this influences the validity of

For peer review only

the readings they upload. In addition, we were interested in how experience of self-reported measurement and accurate reporting might be facilitated by a digital interface. Our research question was 'How do users of a 'digital-only' contraceptive service provide biometric measurements and what does this teach us about safe and effective online care?

Intervention

We studied user experience within a free, online service providing the COCP within a diverse area of South East London, United Kingdom with high rates of socio-economic deprivation and poor sexual and reproductive health indicators, such as high rates of EC use and under-18 conceptions[25].

The service was advertised through social media, print media (cards and stickers) and local clinics. The digital interface of the online service invites users to order the COCP after completing a medical history mapped to national guidelines[21] and report their height, weight and BP. Users are informed that a BP reading is required at the start of the order journey and basic information on the risk of taking the COCP with high BP is provided (appendix A). Those who have ordered the COCP from the service before are reminded two weeks prior to the end of their pill supply to obtain a blood pressure reading in advance of their next order (footnote 1). Users who upload a blood pressure reading outside the normal range are contacted by the prescribing clinician for further details on where and how it was measured and to request additional measurements if appropriate. Users and clinicians communicate mainly be text message and the tone of the clinical communication is neutral and polite for example, 'Hello, thank you for your order, your contraception will be dispatched today, to this address by Royal Mail tracked but not signed for, many thanks, SH:24'.

Contraindications to the COCP are classified within national and international guidelines on a scale of 1 to 4. A contraindication classified as 1 or 2 suggests that the benefits of use outweighs the risks and a contraindication classified as 3 or 4 suggests that the risks outweigh the benefits[21]. The COCP is not prescribed within this service to women reporting category 3 or 4 contraindications. Prescriptions, once authorized by a UK General Medical Council registered doctor are sent electronically to a UK registered online pharmacy which dispenses the medication and posts it to the service user. The service is registered with the Care Quality Commission and is compliant with NHS digital standards.

Methods

Ethical approval for the study was granted by the East Midlands – Leicester Research Ethics Committee (ref 17/EM/0181) and all study participants provided informed consent.

The study used existing, nationally significant PPI conducted by the James Lind Alliance and endorsed by the UK Faculty of Sexual and Reproductive Health Care which found that among the general population 'research to evaluate which interventions increase uptake and continuation rates of effective contraceptive methods' was the number one priority for academic research on contraception in the UK (https://www.fsrh.org/about-us/fsrh-committees/contraception-priority-setting-partnership-psp-in-conjunction/). In addition, we completed our own research priority setting exercise prior to developing the research proposal based on two focus groups with service users that identified research into online

For peer review only

service delivery as a priority topic for research on contraception. The logic model for the intervention was developed based on interviews with 21 stakeholders including clinicians, managers, commissioners and users. Intervention development involved over 100 service users through a process of human centred design. The service designers conducted interviews in clinical settings, observations of clinical consultations, an online questionnaire to inform early prototypes and then tested emerging prototypes with users in clinics. Semi-structured interviews were completed with a purposive sample of 20 participants who had been recruited to a larger study which aimed to understand the similarity between self-reported and researcher-measured BP, height and weight measurements among 365 women who requested COCP from the online service. Participants were recruited to the larger study through an online invitation after they had made an order for the COCP from the online service. From these participants, a purposive sample was selected in order to include participants who had ordered the COCP but were or were not prescribed it, and to include participants from a range of ages.

The semi-structured interviews with participants were completed by HM, a White British, 26-year-old female researcher, who has used COCP. The interview guide started with general questions on first and subsequent experience of accessing contraception, followed by a detailed account of use of the online service with a particular focus on experience of measuring and reporting height, weight and BP. Interviews were completed in a private room in a university building and lasted between 23 and 83 minutes with a median length of 40 minutes and were audio-recorded and transcribed verbatim.

Analysis

We completed an inductive, thematic analysis assisted by NVivo qualitative analysis software (version 12). We read and re-read the interviews (PB, HM, AM) to develop themes and completed an iterative process of four rounds of theme refinement, re-checking our data for material that refuted, supported or developed our themes and re-coding with discussion among the whole research team to reach consensus at each stage. Our final coding strategy is provided in appendix B).

Results

Table 1 shows the sociodemographic characteristics of the study population and the nested qualitative study population. Our respondents were aged between 19 and 37 years and most had participated in higher education.

Table 1: Socio-demographic characteristics and self-reported contraceptive history of participants.

	Study participants n = 365, %	Nested qualitative sample n = 20
**Age (years) mean**	24.76	24.89
18-19	29 (7.95)	2
20-24	175 (47.95)	13
25-34	147 (40.27)	2
35+	14 (8.84)	3
**Ethnicity**		

For peer review only

White English/ Welsh/ Scottish/ Northern Irish/British/ Other	221 (60.55)	12
Black African/ Caribbean/ British/ Other	57 (15.62)	5
Asian/ Asian British	33 (9.04)	0
Mixed/ multiple ethnic groups	42 (11.51)	2
Other ethnic groups	10 (2.74)	1
Not known/ prefer not to say	2 (0.55)	0
**Index of Multiple Deprivation of Postcode of residence**		
1 (most deprived)	131 (36.19)	4
2	148 (40.88)	9
3	66 (18.23)	5
4	13 (3.59)	2
5 (least deprived)	4 (1.10)	0
**Qualifications**		
No academic qualifications	1 (0.27)	0
GCSES (or equivalent level)	13 (3.56)	1
AS/A Levels (or equivalent level)	34 (9.32)	2
Higher education qualifications (or equivalent level)	316 (86.58)	17
Not sure or other	1 (0.27)	0

Our participants reported difficulty accessing the COCP from face-to-face services, were concerned that a gap in their pill supply would put them at risk of pregnancy and were relieved to find that a free online service existed. This added a sense of urgency to their need to access contraception.

> *"Say it was like Friday, I needed the pill by the Monday so I was like panicking, what shall I do? Where do I go now that I tried everywhere and nowhere seemed available? So, yeah, I just looked online like and then just found that you could order it online…… I felt relieved that I could actually get it"*
>
> Participant F, 19-year-old repeat COCP user, prescribed by online service

In a context where they felt access to contraception was difficult they employed a range of strategies within both online and face-to-face care to mitigate this.

> *'Basically, I needed to get a top-up for more pills …..Because I finish work at 6:00 and I start at 9:00, is the perfect hours for not being able to go to a clinic. My sister finishes work at four o'clock. I asked my sister, "Can you get me a top-up please and I'll pay you because I really need it." Like, I'll pay her for her time having to wait in the clinic'.*
>
> Participant C, 20-year-old repeat COCP user, prescribed by online service

Participants felt that the COCP was a low risk medication that was appropriately accessed online. However, as they engaged with the ordering process, the work to measure BP, but not the work to measure height and weight, or report medical history, was seen as a significant barrier to access.

For peer review only

> *"With your blood pressure, there's just no way to tell so it's just very different to someone saying, 'What's your height? What's your weight?' because you can sort of look at yourself in the mirror and guess, but you just can't do that with your blood pressure."*
> Participant D, 24-year-old repeat COCP user, prescribed by online service

Although most users uploaded a BP measurement, they lacked information on normal values, variation over time or the relationship of BP to feeling healthy or stressed. It was clear that repeated use of face-to-face services to obtain the COCP, including regular BP measurements, often over many years, had not provided sufficient knowledge about BP or its relation to COCP use to understand a specific BP measurement although it did provide users with a sense of whether their BP was within the normal range.

> *" like when you go to the clinic to take your blood pressure, no-one tells you what it is. They just take it and then they write it down. No-one says, 'Oh, for your information, your blood pressure's this'. Even if you're like high, you would normally have to say, 'Oh, what was my high?'*
> Participant D, 24-year-old repeat COCP user, prescribed by online service

Although the recommendation from the online service was to obtain a BP measurement at a pharmacy most participants did not recall this advice or follow it except one who was friends with her local pharmacist and obtained her BP reading from this source. The need to visit a clinical setting for a BP check was seen as significantly reducing the convenience of the service.

> *"For me, that kind of takes away the purpose of the service, because I want to use the service to avoid having to go to the pharmacy, because I just want to be quick and do it....."*
> Participant M, 25-year-old repeat COCP user, was not prescribed by online service

Participants felt that the work to obtain a BP measurement was insufficiently acknowledged by the digital interface, while the work to access height and weight measurements was acceptable and required no further support. Social networks were an important source of access to BP machines, for example, use of a machine owned by an older relative or friend or using existing health care professional contacts within social networks. Research was required to understand BP and its measurement. This was mainly online, for example searching for normal BP values and researching the health consequences of high BP. Although service users were advised to obtain a blood pressure reading from their local pharmacy most did not feel that this was a useful option for them. Many participants did not have access to a BP monitor and combined strategies to generate what they felt was an accurate measurement, increasing their confidence in the value they uploaded by triangulating between different methods of measurement. Their accounts of this (see table 2) illustrate the disjuncture between the expectations from the online service, which required the systolic and diastolic readings, and the experience of users, who often knew that their BP was normal but lacked access to the specific readings.

For peer review only

Table 2: Work required to generate a BP measurement though a combination of strategies

Case details	Measurement strategy	Account of the measurement process
Participant F, 19-year-old repeat COCP user, prescribed by online service	Remembered an old reading, knew that BP was usually normal and planned to get it checked before the next order	*"I think that was like from a doctor's that I had, like I think a few months beforehand. So, it was within normal range so it was fine and just remembered it …. so I knew that it wouldn't be like as reliable as doing a blood pressure straight away but within that, like I needed it quickly so I knew that normally like I'm fine with blood pressure so I just put in what I could remember from the last one, like it was normal …. So I think, if I was to like order again, I think I would maybe consider actually taking it like closer to the point"*
Participant O, 22-year-old repeat user of the pill, was not prescribed COCP by online service	Remembered that a previous reading had been normal and looked up a normal value online	*"Yeah, when the blood pressure thing came up I was a bit nervous … I knew that I had healthy blood pressure, so I could kind of estimate… for me I just sort of, googled what a healthy kind of reading was and vaguely remembered from my last time, anyway"*
Participant T, 23-year-old first-time COCP user, prescribed by online service	Estimated BP based on multiple previous readings from GP and home BP machine	*"So, the blood pressure I used an estimate of what the previous readings I'd had in the last twelve months. I'd had a GP visit and I had it checked. And then I'd had it checked a few times at home, 'cause we had a blood pressure monitor at home, that we were trying out and seeing if it worked or not. And all of those had been fine in the past. So I was fairly confident that it wouldn't have changed"*

As a result of the difficulty accessing BP measurements many participants found themselves negotiating their relationship with this new type of clinical service as they sought to understand whether this was a relationship of trust that required honest reporting of biometric measurements. They reflected on the whether their information would be reviewed by a clinician or a digital algorithm. They felt that communication with a clinician would require greater commitment to providing an accurate reading and they worked to understand what might be a reasonable compromise of measurement techniques to provide such a reading if they did not have access to the recent measurement requested by the service. They saw the need to provide BP as something that was required by the service and therefore a barrier to accessing contraception but also as something that might be important for their own health. Some users took on the new responsibility very seriously and took personal responsibility for providing an accurate measurement.

"I just suddenly had like a fear of god moment, where I was like, if I lie about this and then something does happen maybe they'll turn around and be like "well, you're not supposed to be over this weight or you're blood pressure has got to be this amount"…… things with your health, you've got to take seriously. It makes me nervous. So, even if it is the pill, which 9/10 isn't going to be an issue ….. Because I wasn't- there wasn't a face to it, I felt I really needed to make sure I was doing it right"

Participant L, 24 year old repeat COCP user prescribed by the online service

For peer review only

Others felt that this responsibility was shared with the online service and expected the clinicians/algorithms to identify inaccurate readings and uncover inappropriate strategies to collect them. For example, one participant who had guessed a reading that was biologically impossible and was contacted by a clinician from the service to find out when and how it was measured. She was then surprised and a little angry when her (untrue) explanation was accepted, feeling that the clinician should have been more questioning of the information she gave.

"The first message that came up was like "Oh you will be contacted by a nurse or something. To discuss.... further" …. I got a text message from the nurse saying how did you gather your blood pressure information. And I was like "Oh when I went to the clinic, she wrote it down for me". But I just entered it wrong…. And they said "Oh OK then, that's fine. Thank you. We'll let the nurse know…. I thought it was going to be much more nerve-wrecking, like calling my phone …".

Participant G, 24 year old repeat COCP user prescribed by the online service

This participant was prepared to compromise her health in order to obtain the contraception that she needed.

"Then I just had to google it, I just had to google "What is the correct blood pressure". And then one came up, they gave me a average. And I was like OK. Put that in…"

Participant G, 24 year old repeat COCP user prescribed by the online service

Participants wanted more information about BP and its importance in relation to the COCP and more help with obtaining a reading although none of the participants reported requesting help from the service. The digital interface seemed to communicate the BP measurement as the users' responsibility and the inputting of normal measurements as requirement. Participants felt that making contact might compromise their entitlement to the COCP and none did so unless the service contacted them first.

Throughout the interviews there was a marked difference between participants' description of measuring BP and their experience of measuring height and weight. They felt that monitoring their weight was a common health maintenance activity and they had been previously encouraged to do this. Height does not change in adulthood and most people have had it measured at some time and remember this measurement at least approximately.

Discussion

The need to measure BP was experienced as an important barrier to access an otherwise convenient online contraceptive service. Significant work was required by users, the digital interface and clinicians to facilitate the process of BP measurement, communication and assessment (figure 1). Users completed social, ethical and information gathering work to provide numerical BP values often combining strategies to maximise the validity of the reading uploaded, researching what is normal and negotiating what they felt was an appropriate commitment to providing accurate information. This negotiation referenced their need to access the pill urgently, feelings about a trusting relationship with the clinical

service and concerns about the health implications of taking the pill with high BP. Better communication of the presence of the clinicians within the service and their willingness to support users would have made this process easier. Users found the measurement of height and weight straightforward and did not experience this as a barrier to access the service in any way.

Developing capacity to utilise effective, evidence based self-care for sexual and reproductive health is a public good[1] and may expand access to contraception[20], abortion[26] and testing for sexually transmitted infections[5]. However, self-care within health systems involves delegation and re-distribution of health work so that while it may allow people greater control over their lives it also brings new responsibilities[27] and new requirements for clinical support.

Whereas health policy discourses have tended to portray the digital interfaces that mediate digital health care as neutral channels with predictable outcomes[28], this study and others[15, 29] suggest that digital interfaces do important work in brokering (or not) relationships of trust within online clinical services and that these will influence user experience and clinical outcomes[28, 30]. New media for consultations inevitably change some aspects of clinical relationships[31] and at present very little is known about the 'rules of engagement' for the management of clinical consultations through online portals[16, 32]. Our study suggests that it is important that both user and provider are clearly present as individuals within the online communication process, that provider commitment to building a clinical relationship is clearly demonstrated and visible to the service user and that the health benefits of accurate reporting of biometric measurements are clear.

Self-monitoring blood pressure has become popular among those with long term conditions and GPs estimate that almost a third of patients with hypertension self-monitor their blood pressure[33]. Similarly, research among pregnant women with gestational hypertension shows that self-measurement with support and training is acceptable and empowering[34-35]. However, experience of self- measurement of blood pressure for the population of young contraceptive users in our study seems to be quite different as their need to self-measure is intermittent, they lack information and received no training on BP and its measurement.

Conclusions

Our findings support the view that digitally facilitated self-management will require close collaboration between users and providers, particularly in 'digital-first' services. The digital interface is a particularly important in communicating and supporting this collaboration and its importance is often underestimated. As digitally facilitated self-management becomes increasingly prevalent within health systems we suggest that the digital interfaces that support them should:

1. Fully acknowledge the work that users, providers and digital interfaces do within online services.
2. Ensure that user and provider are clearly present as individuals within the online communication process, that provider commitment to building a clinical relationship

For peer review only

is demonstrated and that the health benefits of the provision of accurate information are communicated.

3. Enable appropriate adaptation of clinical processes outside clinical contexts to facilitate self-care, for example asking whether BP was normal rather than asking for specific values.

The service evaluated is currently being re-designed in response to this research aiming to communicate better information about BP and its measurement, realistic strategies for BP measurement and a commitment to collaborative user/provider relationship.

Footnote 1: text message sent to users to remind them to check their blood pressure before they order their next supply of pills
You have 2 weeks supply left of your oral contraception. You can repeat order here: https://sh24.org.uk/coc_orders/new To successfully repeat you will need to know blood pressure. IF YOU DO NOT KNOW YOUR BLOOD PRESSURE you can find out how to check it here: https://sh24.org.uk/contraception/order#question_7 Text back if you would like help. Thanks, SH:24

Funding
This report is independent research funded by the National Institute for Health Research (Research for Patient Benefit Programme, PB-PG-0815-20009). The views expressed in this publication are those of the author(s) and not necessarily those of the NIHR or the Department for Health and Social Care

Competing interests
Paula Baraitser is clinical director of the online service studied and is one of the prescribers within this service. Since completion of this study, Hannah McCulloch has worked at the online service on a different research project. This work is part of a process of research on innovation at SH:24 where all innovation is the subject of research to ensure that learning is shared widely. SH:24 is a 'not for profit' organisation that provides health services to the National Health Service and sharing learning through innovation and research is one of the principles of the organisation.

Author statement
Paula Baraitser contributed to the conception and design of the study, oversaw data collection, contributed to data analysis and drafted the manuscript.
Hannah McCulloch contributed to data collection and analysis and contributed to the manuscript on the manuscript.
Alessandra Morelli contributed to data collection and analysis and commented on the manuscript
Caroline Free contributed to the conception and design of the study, contributed to data analysis and commented on the manuscript.

Data statement
The anonymised qualitative interviews are available from the authors on request.

References

For peer review only

1. Narasimhan M., Allotey P., Hardon A., Self-care interventions to advance health and wellbeing: a conceptual framework to inform normative guidance, British Medical Journal, 2019;365.

2. Armstrong. D. Actors, patients and agency: a recent history. Sociology of Illness and Health, 2014; 36: 163-174.

3. Lorig K., Sobel D., Stewart A., Brown B., Bandura A., Ritter P., Gonzalez V., Laurent D., Holman H. Evidence suggesting that a chronic disease self-management programme can improve health status while reducing hospitalization: a randomized controlled trial. Medical Care, 1999; 37: 5-14.

4. Mol, A. 'Living with Diabetes: Care Beyond Choice and Control.' The Lancet 2009; 373: 1756– 7.

5. Wilson W., Morris T., Syred J., Menon-Johansson A., Palmer M., Barnard S., Rezel E., Baraitser P. e-STI testing and results service: a single blind randomised controlled trial. Plos Medicine, 2017; 14: e1002479.

6. Eysenbach, G. What is e-health? Journal of Medical Internet Research, 2001; 3: e20.

7. Sheikh A., Sood H., Bates D. Leveraging health information technology to achieve the "triple aim" of healthcare reform. J Am Med Inform Assoc 2015; 4: 849-56.

8. World Health Organisation Guideline: Recommendations on digital interventions for health system strengthening, 2019 https://www.who.int/reproductivehealth/publications/digital-interventions-health-system-strengthening/en/

9. NHS digital. Personalised Health and Care 2020. National Information Board and Department of Health and Social Care, 2014. https://www.gov.uk/government/publicati§ons/personalised-health-and-care-2020 (Accessed 23/01/2020)

10. Lupton, Deborah, Self-Tracking Modes: Reflexive Self-Monitoring and Data Practices Paper for the 'Imminent Citizenships: Personhood and Identity Politics in the Informatic Age' workshop, 27 August 2014, ANU, Canberra http://dx.doi.org/10.2139/ssrn.2483549

11. Piras E. and Miele F.Clinical self-tracking and monitoring technologies: negotiations in the ICT-mediated patient–provider relationship, Health Sociology Review, 2017; 26: 38-53.

12. Greenhalgh, T., J. Wherton, P. Sugarhood, S. Hinder, R. Procter, and R. Stones. 2013. ' What Matters to Older People with Assisted Living Needs? A Phenomenological Analysis of the Use and Non-Use of Telehealth and Telecare.' Social Science & Medicine 93: 86– 95.

13. May C., Finch T., Mair F., Mort M. Towards a wireless patient: chronic illness, scarce care and technological innovation in the United Kingdom. Social Science and Medicine, 2005; 61: 1485-94.

14. Mort, M., Finch T., May C. ‘ Making and Unmaking Telepatients: Identity and Governance in New Health Technologies.’ Science, Technology & Human Values,2009; 34: 9–33.

15. Mort, M., Roberts C., Callén B. ‘ Ageing with Telecare: Care or Coercion in Austerity?’ Sociology of Health & Illnes, 2013; 35: 799–812.

16. Rodgers M., Raine G., Thomas S. Informing NHS Policy in ‘digital-first’ primary care: a rapid evidence synthesis. Health Services and Delivery Research, 2019: 1-54 ISSN 2050-4357.

17. Baraitser P. and Cribb A. ‘Putting people in charge of their own health and care: Using meta-narrative review and the example of sexual health services to re-think relationships between e-health and agency. Health Expectations, 2019, 22; 5: 838-848.

18. Turner K., Looker K., Syred J., Zienkiewicz A., Baraitser P. Online testing for sexually transmitted infections: a whole systems approach to predicting value. PLoS One. 2019, 14(2): e0212420.

19. Hogenson, Eiluned & Jett-Goheen, Mary & Gaydos, Charlotte. (2019). An Analysis of User Survey Data for an Internet Program for Testing for Sexually Transmitted Infections, I Want the Kit (IWTK) in Maryland and Washington D.C. Sexually transmitted diseases, 2019; 46: 768-770.

20. Rezel-Potts. E., Palmer M., Free C., Baraitser P., The service users of online contraception: a cohort study. The Lancet, 2019, 394, S80.

21. FSRH, Faculty of Sexual and Reproductive Health Care UK Medical Eligibility Criteria for Contraceptive Use, 2016. https://www.fsrh.org/standards-and-guidance/uk-medical-eligibility-criteria-for-contraceptive-use-ukmec/ (accessed 27/11/2019)

22. World Health Organisation. Medical Eligibility Criteria for Contraceptive Use, 5th Edition, 2015. https://www.who.int/reproductivehealth/publications/family_planning/Ex-Summ-MEC-5/en/ (accessed 28/11/2019)

23. Oelkers WK. Effects of estrogens and progestogens on the renin-aldosterone system and blood pressure. Steroids. 1996;61(4):166–171.

24. Faculty of Sexual and Reproductive Health, Clinical Guidance on Combined Hormonal Methods of Contraception, 2019. https://www.fsrh.org/standards-and-guidance/documents/combined-hormonal-contraception/ (accessed 27/11/2019).

25. Lambeth, Southwark and Lewisham Public Health departments, Sexual and Reproductive Health Strategy 2019-2024, 2019, https://www.lambeth.gov.uk/sites/default/files/yh-lsl-sexual-health-strategy_0.pdf (accessed 20/11/2019)

26. Aiken A., Digol I., Trussel J., Gomperts R. Self reported outcomes and adverse events after medical abortion through online telemedicine population based study in the Republic of Ireland and Northern Ireland BMJ, 2017; 357:j2011

27. Lupton D. 'Quantified sex: a critical analysis of sexual and reproductive self-tracking using apps', *Culture, Health and Sexuality*, 2015; 17: 440 -453.

28. Lupton D. Towards sensory studies of digital health. Digital Health, 2017; 3: 1-6.

29. Pols J., Willems D., Innovation and Evaluation: taming and unleashing health care technology. Sociology of Illness and Health, 2011; 33: 484-498.

30. Fors V. and Pink S. Pedagogy as Possibility: Health Interventions as Digital Openess. Social Sciences, 2017, 6: 59-71.

31. Atherton H and Ziebland S. What do we need to consider when planning, implementing and researching the use of alternatives to face-to-face consultations in primary healthcare? Digital Health, 2016; 2:1-13.

32. Atherton H., Pappas Y., Heneghan C., Murray E. Experiences of using email for general practice consultations: a qualitative study. British Journal of General Practice, 2013; 63: e760-767.

33. McManus RJ, Wood S, Bray EP, et al. Self-monitoring in hypertension: a web-based survey of primary care physicians. Journal of Human Hypertension. 2014; 28: 123–127.

34. Hinton L, Tucker K, Greenfield S, Hodgkinson J, Mackillop L, McCourt C, Carver T, Crawford C, Glogowska M, Locock L, Selwood M, Taylor KS, McManus RJ. Blood pressure self-monitoring in pregnancy (BuMP) feasibility study; a qualitative analysis of women's experiences of self-monitoring. BMC Pregnancy Childbirth, 2017; 19: 17-42.

35. Tucker K., Taylor K., Crawford C., Hodgkinson J., Bankhead C., Carver T., Ewers E., Glogowska M., Greenfield S., Ingram L., Hinton L., Khan K., Locock L., Mackillop L., McCourt C., Pirie A., Stevens R., McManus R. Blood pressure self-monitoring in

For peer review only

pregnancy: Examining feasibility in a prospective cohort study. BMC Pregnancy and Childbirth, 2017; 17: 442-452.

For peer review only

Figure 1: The work done by the user, digital interface and clinician to support communication of accurate biometric measurements.

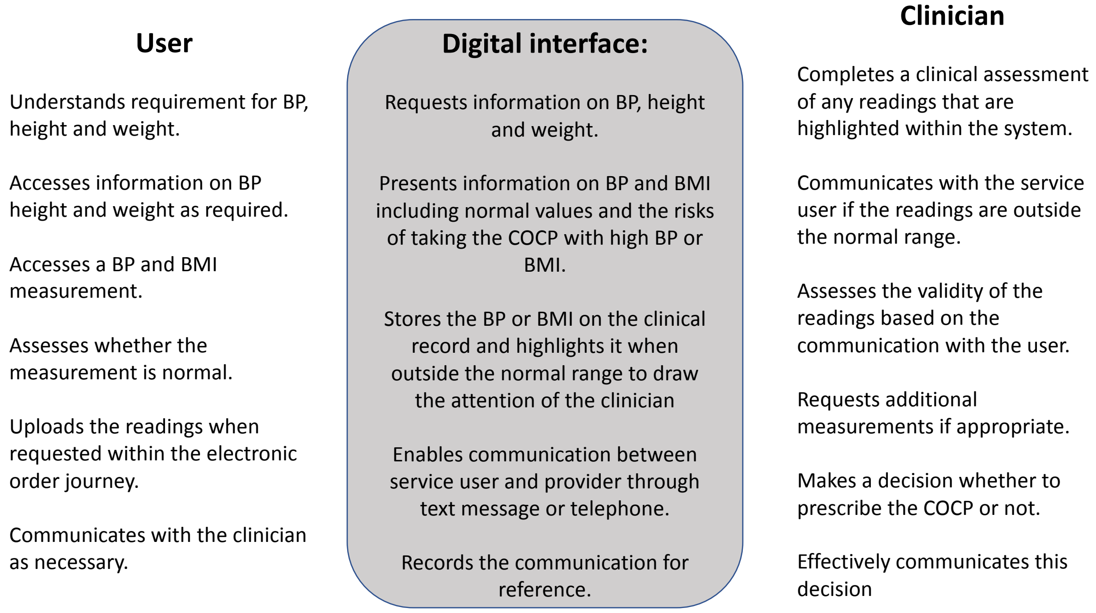

review only

Appendix A – Information provided by the service on the risks of taking the COCP with high blood pressure

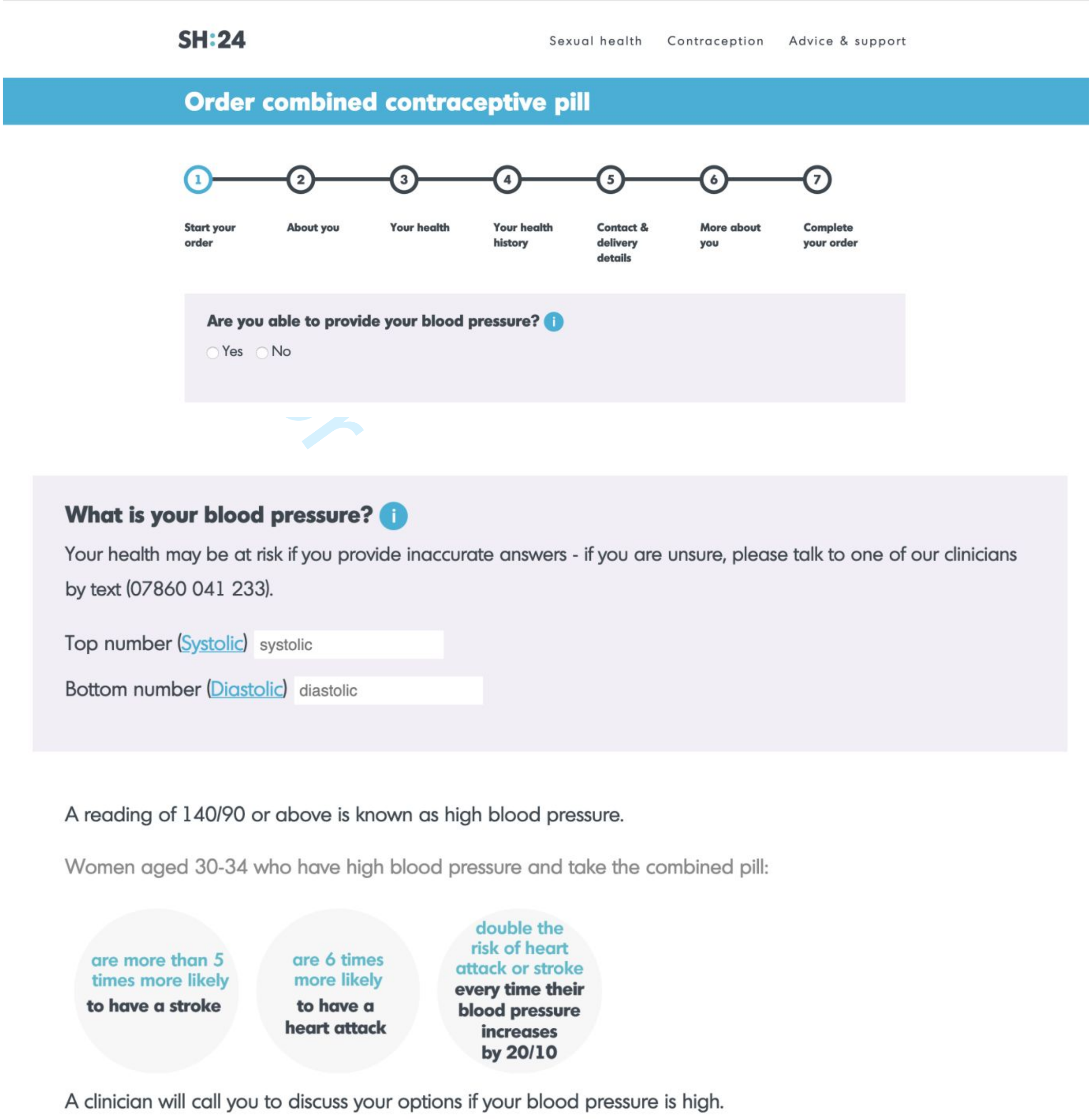

Appendix B: Qualitative paper coding tree

Theme	Sub-themes	Notes
The practical realities of obtaining contraception from traditional services and the choice to order the pill online	An alternative to inconvenient existing options<br><br>Fits into my lifestyle<br><br>A solution to an urgent situation	Navigating access to contraception with time pressure - Time and urgency is of fundamental importance in this assessment. Reference negative case study of person who did not have time pressure
A new contraceptive service option generates new work, new roles and new responsibilities.	Social work (Drawing on existing relationships to access a BP reading)<br><br>Ethical work (Negotiating an ethical position under pressure to obtain contraception)<br><br>Research work (online research to find out what is normal and the consequences of taking the pill with high BP).	By changing the frame of access and the nature of the interface with the health system we have created a new option with a new set of practical realities.<br><br>No existing pathways, so created new pathways, used chance events. Translating past experience into this new context.<br><br>Differences between strategies to measure BP (unfamiliar) and height/weight (familiar)
Ideas about blood pressure and its measurement.	Blood pressure as an intangible concept<br><br>Blood pressure as linked to own health and normality – from past clinical experiences<br><br>BP as a medical thing whereas BMI is more in the public domain.<br><br>Blood pressure and how recent it needs to be?	Consequences of these ideas for choice of measurement strategy.
Responsibility and risk	Negotiating a clinical relationship with a faceless service<br><br>The characteristics of the digital interface that supported or did not support effective communication.	

For peer review only

Reporting checklist for qualitative study.

Based on the SRQR guidelines.

Instructions to authors

Complete this checklist by entering the page numbers from your manuscript where readers will find each of the items listed below.

Your article may not currently address all the items on the checklist. Please modify your text to include the missing information. If you are certain that an item does not apply, please write "n/a" and provide a short explanation.

Upload your completed checklist as an extra file when you submit to a journal.

In your methods section, say that you used the SRQRreporting guidelines, and cite them as:

O'Brien BC, Harris IB, Beckman TJ, Reed DA, Cook DA. Standards for reporting qualitative research: a synthesis of recommendations. Acad Med. 2014;89(9):1245-1251.

		Reporting Item	Page Number
**Title**			
	#1	Concise description of the nature and topic of the study identifying the study as qualitative or indicating the approach (e.g. ethnography, grounded theory) or data collection methods (e.g. interview, focus group) is recommended	1
**Abstract**			
	#2	Summary of the key elements of the study using the abstract format of the intended publication; typically includes background, purpose, methods, results and conclusions	1
**Introduction**			
Problem formulation	#3	Description and signifcance of the problem / phenomenon studied: review of relevant theory and empirical work; problem statement	2

For peer review only

Purpose or research question	#4	Purpose of the study and specific objectives or questions	1 and 3
**Methods**			
Qualitative approach and research paradigm	#5	Qualitative approach (e.g. ethnography, grounded theory, case study, phenomenolgy, narrative research) and guiding theory if appropriate; identifying the research paradigm (e.g. postpositivist, constructivist / interpretivist) is also recommended; rationale. The rationale should briefly discuss the justification for choosing that theory, approach, method or technique rather than other options available; the assumptions and limitations implicit in those choices and how those choices influence study conclusions and transferability. As appropriate the rationale for several items might be discussed together.	4-5
Researcher characteristics and reflexivity	#6	Researchers' characteristics that may influence the research, including personal attributes, qualifications / experience, relationship with participants, assumptions and / or presuppositions; potential or actual interaction between researchers' characteristics and the research questions, approach, methods, results and / or transferability	4 and 11
Context	#7	Setting / site and salient contextual factors; rationale	4
Sampling strategy	#8	How and why research participants, documents, or events were selected; criteria for deciding when no further sampling was necessary (e.g. sampling saturation); rationale	4
Ethical issues pertaining to human subjects	#9	Documentation of approval by an appropriate ethics review board and participant consent, or explanation for lack thereof; other confidentiality and data security issues	4
Data collection methods	#10	Types of data collected; details of data collection procedures including (as appropriate) start and stop dates of data collection and analysis, iterative	4-5

For peer review only

		process, triangulation of sources / methods, and modification of procedures in response to evolving study findings; rationale	
Data collection instruments and technologies	#11	Description of instruments (e.g. interview guides, questionnaires) and devices (e.g. audio recorders) used for data collection; if / how the instruments(s) changed over the course of the study	4-5
Units of study	#12	Number and relevant characteristics of participants, documents, or events included in the study; level of participation (could be reported in results)	5
Data processing	#13	Methods for processing data prior to and during analysis, including transcription, data entry, data management and security, verification of data integrity, data coding, and anonymisation / deidentification of excerpts	5
Data analysis	#14	Process by which inferences, themes, etc. were identified and developed, including the researchers involved in data analysis; usually references a specific paradigm or approach; rationale	5
Techniques to enhance trustworthiness	#15	Techniques to enhance trustworthiness and credibility of data analysis (e.g. member checking, audit trail, triangulation); rationale	4
**Results/findings**			
Syntheses and interpretation	#16	Main findings (e.g. interpretations, inferences, and themes); might include development of a theory or model, or integration with prior research or theory	5-9
Links to empirical data	#17	Evidence (e.g. quotes, field notes, text excerpts, photographs) to substantiate analytic findings	5-9
**Discussion**			
Intergration with prior work, implications, transferability and contribution(s) to the field	#18	Short summary of main findings; explanation of how findings and conclusions connect to, support, elaborate on, or challenge conclusions of earlier scholarship; discussion of scope of application /	9-10

For peer review only

		generalizability; identification of unique contributions(s) to scholarship in a discipline or field	
Limitations	#19	Trustworthiness and limitations of findings	2
**Other**			
Conflicts of interest	#20	Potential sources of influence of perceived influence on study conduct and conclusions; how these were managed	11
Funding	#21	Sources of funding and other support; role of funders in data collection, interpretation and reporting	11

The SRQR checklist is distributed with permission of Wolters Kluwer © 2014 by the Association of American Medical Colleges. This checklist was completed on 17. February 2020 using https://www.goodreports.org/, a tool made by the EQUATOR Network in collaboration with Penelope.ai

For peer review only

BMJ Open

How do users of a 'digital-only' contraceptive service provide biometric measurements and what does this teach us about safe and effective online care? A qualitative interview study

Journal:	*BMJ Open*
Manuscript ID	bmjopen-2020-037851.R1
Article Type:	Original research
Date Submitted by the Author:	02-Jul-2020

Complete List of Authors:	Baraitser, Paula; SH:24<br>McCulloch, Hannah; King's College London, King's Centre for Global Health and Health Partnerships<br>Morelli, Alessandra; King's College London, King's Centre for Global Health and Health Partnerships<br>Free, Caroline; London School of Hygiene and Tropical Medicine, Public Health interventions Unit

**Primary Subject Heading**:	Sexual health
Secondary Subject Heading:	Health services research, Sexual health, Qualitative research, Patient-centred medicine
Keywords:	Telemedicine < BIOTECHNOLOGY & BIOINFORMATICS, Quality in health care < HEALTH SERVICES ADMINISTRATION & MANAGEMENT, SEXUAL MEDICINE

SCHOLARONE™
Manuscripts

I, the Submitting Author has the right to grant and does grant on behalf of all authors of the Work (as defined in the below author licence), an exclusive licence and/or a non-exclusive licence for contributions from authors who are: i) UK Crown employees; ii) where BMJ has agreed a CC-BY licence shall apply, and/or iii) in accordance with the terms applicable for US Federal Government officers or employees acting as part of their official duties; on a worldwide, perpetual, irrevocable, royalty-free basis to BMJ Publishing Group Ltd ("BMJ") its licensees and where the relevant Journal is co-owned by BMJ to the co-owners of the Journal, to publish the Work in this journal and any other BMJ products and to exploit all rights, as set out in our licence.

The Submitting Author accepts and understands that any supply made under these terms is made by BMJ to the Submitting Author unless you are acting as an employee on behalf of your employer or a postgraduate student of an affiliated institution which is paying any applicable article publishing charge ("APC") for Open Access articles. Where the Submitting Author wishes to make the Work available on an Open Access basis (and intends to pay the relevant APC), the terms of reuse of such Open Access shall be governed by a Creative Commons licence – details of these licences and which Creative Commons licence will apply to this Work are set out in our licence referred to above.

Other than as permitted in any relevant BMJ Author's Self Archiving Policies, I confirm this Work has not been accepted for publication elsewhere, is not being considered for publication elsewhere and does not duplicate material already published. I confirm all authors consent to publication of this Work and authorise the granting of this licence.

For peer review only

How do users of a 'digital-only' contraceptive service provide biometric measurements and what does this teach us about safe and effective online care? A qualitative interview study

Paula Baraitser, Hannah McCulloch, Alessandra Morelli, Caroline Free

Paula Baraitser, Paula_baraitser@mac.com (corresponding author) Paula Baraitser, SH:24 35A Westminster Bridge Road, London, SE1 7JB, +442076202250

Hannah MCulloch, Kings Centre for Global Health and Health Partnerships, King's College London, London, UK hannah.mcculloch@kcl.ac.uk

Alessaandra Morelli, King's Centre for Global Health and Health Parterships, King's College London, London, UK King's College London, alessandra.morelli@kcl.ac.uk

Caroline Free, Department of Population Health, London School of Hygiene and Tropical Medicine, London, UK Caroline.Free@lshtm.ac.uk

Word count: 3090

Key words: digital health, sexual health, contraception, blood pressure

ABSTRACT

Objectives

- To describe user experience of obtaining and uploading biometric measurements to a 'digital only' contraceptive service prior to a prescription for the combined oral contraceptive pill.
- To analyse this experience to inform the design of safe and acceptable 'digital only' online contraceptive services.

Setting

An online contraceptive service available free of charge to women in South East London, United Kingdom.

Participants

Twenty participants who had ordered the COCP online. Our purposive sampling strategy ensured that we included participants from a wide range of ages and those who were and were not prescribed the COCP.

Intervention

A 'digital only' contraceptive service that prescribes the combined oral contraceptive pill after an online medical history and self-reported height, weight and blood pressure with pills prescribed by a GMC registered doctor, dispensed by an online pharmacy and posted to the user.

Design

For peer review only

Semi-structured interviews with a purposive sample of 20 participants who were already enrolled in a larger study of this service.

Analysis
Inductive, thematic analysis of the interviews assisted by NVivo qualitative analysis software.

Results
Users valued the convenience of 'digital-only care' but experienced measuring blood pressure but not height or weight as a significant barrier to service use. They actively engaged in work to understand and measure blood pressure through a combination of recent/past measurements, borrowed machines, health service visits and online research. They negotiated tensions around maintaining a trusting relationship with the service, meeting its demands for accurate information while also obtaining the contraception that they needed.

Conclusion
Digital strategies to build trusting clinical relationships despite a lack of face-to-face contact are needed in 'digital only' health services. This includes acknowledgement of work required, evidence of credible human support and a digital interface that communicates the health benefits of collaborating with an engaged clinical team.

STRENGTHS AND LIMITATIONS OF THIS STUDY

This is the first study that looks at user experience of online contraceptive access.

Through qualitative interviews it provides detailed accounts of the work required to negotiate a relationship with a 'digital-only' service.

The analysis identifies elements of user relationships with digital services that support accurate reporting of biometric measurements.

Like all qualitative research it offers in-depth accounts from small numbers of people and may not be representative of larger populations.

Background
People have always acted to maintain and improve their own health, but self-care within formal health systems is a recent development[1-2]. Early research and interventions on self-care within clinical systems focused on shared decision making and symptom management for those living with long term conditions[2-3]. More recently, complex self-care tasks, for example, blood sugar monitoring[4] or self-testing for sexually transmitted infections[5] are included. These tasks often happen outside clinical contexts with remote clinical support and require access to clinical technologies (such as blood sugar monitors or lancets to collect blood) and digital interfaces that support technology use by storing data, providing information and facilitating remote communication between users and clinicians.

For peer review only

National and international health policy documents suggest that digitally enabled, self-care offers the potential to improve user experience with more convenient, more informative and more cost-effective services[6-9]. There is a growing body of literature on digital health services that support home self-monitoring of those with long term conditions such as diabetes or hypertension as an adjunct to health care from a face-to-face service[10-11] and on digital self-monitoring of biometric data (self-tracking) that occurs outside the health system. This literature suggests that outcomes of transferring clinical technologies to domestic spaces may be unpredictable as they are adapted by users for home settings, and framed within non-clinical understandings of health and health care[10-15]. There is less research on digitally facilitated self-care as a stand-alone intervention without a 'face-to-face' element[16]. This gap in the evidence has implications for the 'digital only' or 'digital first' services that are increasingly part of health policy[9].

Sexual health services have been early adopters of digitally enabled self-care[17], possibly because self-care is particularly attractive where stigma is a barrier to service access[1]. Online testing for sexually transmitted infection is now routine practice in many public health systems[5, 18, 19] and there is an expanding market for online contraception[20]. These services are 'online only' services where it is anticipated that the whole clinical pathway will be delivered online.

We studied a UK based, online sexual health service providing the combined oral contraceptive pill (COCP), delivered home, by post after completion of a medical history and self-reporting of height, weight and blood pressure (BP). As an early example of 'digital only' facilitated self-care, we were interested in user experience of measuring and uploading BP, height and weight readings prior to online prescriptions. This is an example of 'pushed' self-tracking[10] or 'clinical self-tracking[11], where the service requires self-measurement as a condition of service delivery.

The measurement of BP and body mass index (BMI) prior to first prescription of COCP is recommended by national and international guidance[21-22]. Ethinyl oestradiol, (the oestrogenic component of most COCPs) stimulates an increase in hepatic protein production including angiotensinogen leading to a slightly reduced renal blood flow and therefore a slight increase in BP[23]. In about 2% of women BP may increase significantly after starting the COCP[24]. Since hypertension increases risk of cardiovascular disease, BP is monitored before first prescription of the pill and then annually and the COCP is contraindicated if BP is consistently above 140/90 mmHg. Obesity is similarly associated with risk of cardiovascular disease, and height and weight are also measured prior to COCP prescription. If BMI (weight in kilograms divided by height squared in metres) is equal to or greater than 35kg/m^2 then the risks of taking the pill are considered to outweigh the benefits[21]. In face-to-face clinical practice, biometric measurements are collected and recorded by health care professionals prior to prescribing, usually as a single measurement with repeat readings taken if the first reading is abnormal. In the online service users are asked to upload a recent reading.

Accuracy of biometric readings in this context is clinically important and may be influenced by user experience of this process[10]. We were therefore interested in how users experience the process of self-reported measurement and how this influences the validity of

For peer review only

the readings they upload. In addition, we were interested in how experience of self-reported measurement and accurate reporting might be facilitated by a digital interface. Our research question was 'How do users of a 'digital-only' contraceptive service provide biometric measurements and what does this teach us about safe and effective online care?

Intervention

We studied user experience within a free, online service providing the COCP within a diverse area of South East London, United Kingdom with high rates of socio-economic deprivation and poor sexual and reproductive health indicators, such as high rates of EC use and under-18 conceptions[25].

The service was advertised through social media, print media (cards and stickers) and local clinics. The digital interface of the online service invites users to order the COCP after completing a medical history mapped to national guidelines[21] and report their height, weight and BP. Users are informed that a BP reading is required at the start of the order journey and basic information on the risk of taking the COCP with high BP is provided (appendix A). The service provides advice on how to obtain a blood pressure measurement from the machines available in general practice waiting rooms and pharmacies or by using a home blood pressure machine. Those who have ordered the COCP from the service before are reminded two weeks prior to the end of their pill supply to obtain a blood pressure reading in advance of their next order (footnote 1). Users who upload a blood pressure reading outside the normal range are contacted by the prescribing clinician for further details on where and how it was measured and to request additional measurements if appropriate. Users and clinicians communicate mainly be text message and the tone of the clinical communication is neutral and polite for example, 'Hello, thank you for your order, your contraception will be dispatched today, to this address by Royal Mail tracked but not signed for, many thanks, SH:24'. Where the prescribing clinician finds that a BP or body mass index that is higher than the levels required for safe prescribing that are specified in national guidance then users are denied the COCP and signposted to information about the alternatives including the option to order the progestagen only pill which does not have the same contraindications. This pathway requires 'work' done by the three entities involved in this process, the service user, the digital interface and the prescribing clinician. This work is described in figure 1.

Insert figure 1 here

Contraindications to the COCP are classified within national and international guidelines on a scale of 1 to 4. A contraindication classified as 1 or 2 suggests that the benefits of use outweigh the risks and a contraindication classified as 3 or 4 suggests that the risks outweigh the benefits[21]. The COCP is not prescribed within this service to women reporting category 3 or 4 contraindications. Prescriptions are authorized by a UK General Medical Council registered doctor are sent electronically to a UK registered online pharmacy which dispenses the medication and posts it to the service user. The service is registered with the Care Quality Commission and is compliant with NHS digital standards.

For peer review only

Methods

Ethical approval for the study was granted by the East Midlands – Leicester Research Ethics Committee (ref 17/EM/0181) and all study participants provided informed consent.

The nested qualitative study reported here aimed to understand user experience of obtaining and uploading biometric measurements prior to online contraceptive prescriptions and was part of a larger study that compared self-reported height, weight and blood pressure (BP) measurements submitted to an online contraceptive service with researcher-measured values.

Semi-structured interviews with participants were completed by HM, a White British, 26-year-old female researcher, who has used COCP. The interview guide started with general questions on first and subsequent experience of accessing contraception, followed by a detailed account of use of the online service with a particular focus on experience of measuring and reporting height, weight and BP. Interviews were completed in a private room in a university building and lasted between 23 and 83 minutes with a median length of 40 minutes and were audio-recorded and transcribed verbatim.

Public and Patient Involvement

Patients and the public were involved in identifying the importance of this research question. We used existing, nationally significant PPI conducted by the James Lind Alliance and endorsed by the UK Faculty of Sexual and Reproductive Health Care which found that among the general population 'research to evaluate which interventions increase uptake and continuation rates of effective contraceptive methods' was the number one priority for academic research on contraception in the UK (https://www.fsrh.org/about-us/fsrh-committees/contraception-priority-setting-partnership-psp-in-conjunction/). In addition, we completed our own research priority setting exercise involving patients and the public prior to developing the research proposal based on two focus groups with service users that identified research into online service delivery as a priority topic for research on contraception. The logic model for the intervention was similarly developed with input from patients and the public including interviews with 21 stakeholdes. Intervention development involved over 100 service users through a process of human centred design.

Analysis

We completed an inductive, thematic analysis assisted by NVivo qualitative analysis software (version 12). We read and re-read the interviews (PB, HM, AM) to develop themes and completed an iterative process of four rounds of theme refinement, re-checking our data for material that refuted, supported or developed our themes and re-coding with discussion among the whole research team to reach consensus at each stage. Our final coding strategy is provided in appendix B.

Results

Table 1 shows the sociodemographic characteristics of the study population and the nested qualitative study population. Our respondents were aged between 19 and 37 years and most had participated in higher education.

For peer review only

Table 1: Socio-demographic characteristics and self-reported contraceptive history of participants.

	Study participants of quantitative study from which the qualitative sample was identified, n = 365 (%)	Study participants of nested qualitative sample, n = 20
**Age (years) mean**	24.76	24.89
18-19	29 (7.95)	2
20-24	175 (47.95)	13
25-34	147 (40.27)	2
35+	14 (8.84)	3
**Ethnicity**		
White English/ Welsh/ Scottish/ Northern Irish/British/ Other	221 (60.55)	12
Black African/ Caribbean/ British/ Other	57 (15.62)	5
Asian/ Asian British	33 (9.04)	0
Mixed/ multiple ethnic groups	42 (11.51)	2
Other ethnic groups	10 (2.74)	1
Not known/ prefer not to say	2 (0.55)	0
**Index of Multiple Deprivation of Postcode of residence**		
1 (most deprived)	131 (36.19)	4
2	148 (40.88)	9
3	66 (18.23)	5
4	13 (3.59)	2
5 (least deprived)	4 (1.10)	0
**Qualifications**		
No academic qualifications	1 (0.27)	0
GCSES (or equivalent level)	13 (3.56)	1
AS/A Levels (or equivalent level)	34 (9.32)	2
Higher education qualifications (or equivalent level)	316 (86.58)	17
Not sure or other	1 (0.27)	0

The themes that emerged during analysis related to the convenience of ‘digital-only care’ but also to the measurement of blood pressure, but not height or weight, as a barrier to service use. Users acknowledged the work required to fulfil the requirements of the service and actively engaged with the need to understand and measure blood pressure. They negotiated tensions around maintaining a trusting relationship with the service, meeting its demands for accurate information while also obtaining the contraception that they needed at the time that they needed it.

Our participants reported difficulty accessing the COCP from face-to-face services, were concerned that a gap in their pill supply would put them at risk of pregnancy and were relieved to find that a free online service existed. This added a sense of urgency to their need to access contraception.

For peer review only

"Say it was like Friday, I needed the pill by the Monday so I was like panicking, what shall I do? Where do I go now that I tried everywhere and nowhere seemed available? So, yeah, I just looked online like and then just found that you could order it online...... I felt relieved that I could actually get it" Participant F

In a context where they felt access to contraception was difficult, they employed a range of strategies within both online and face-to-face care to mitigate this.

'Basically, I needed to get a top-up for more pills ..... Because I finish work at 6:00 and I start at 9:00, is the perfect hours for not being able to go to a clinic. My sister finishes work at four o'clock. I asked my sister, "Can you get me a top-up please and I'll pay you because I really need it." Like, I'll pay her for her time having to wait in the clinic'. Participant C

Participants felt that the COCP was a low risk medication that was appropriately accessed online. However, as they engaged with the ordering process, the work to measure BP, but not the work to measure height and weight, or report medical history, was seen as a significant barrier to access.

"With your blood pressure, there's just no way to tell so it's just very different to someone saying, 'What's your height? What's your weight?' because you can sort of look at yourself in the mirror and guess, but you just can't do that with your blood pressure." Participant D

Although most users uploaded a BP measurement, they lacked information on normal values, variation over time or the relationship of BP to feeling healthy or stressed. It was clear that repeated use of face-to-face services to obtain the COCP, including regular BP measurements, often over many years, had not provided sufficient knowledge about BP or its relation to COCP use to understand a specific BP measurement although it did provide users with a sense of whether their BP was within the normal range.

" like when you go to the clinic to take your blood pressure, no-one tells you what it is. They just take it and then they write it down. No-one says, 'Oh, for your information, your blood pressure's this'. Even if you're like high, you would normally have to say, 'Oh, what was my high?' Participant D

Although the recommendation from the online service was to obtain a BP measurement at a pharmacy most participants did not recall this advice or follow it except one who was friends with her local pharmacist and obtained her BP reading from this source. The need to visit a clinical setting for a BP check was seen as significantly reducing the convenience of the service.

"For me, that kind of takes away the purpose of the service, because I want to use the service to avoid having to go to the pharmacy, because I just want to be quick and do it....." Participant M,

For peer review only

Participants felt that the work to obtain a BP measurement was insufficiently acknowledged by the digital interface, while the work to access height and weight measurements was acceptable and required no further support. Social networks were an important source of access to BP machines, for example, use of a machine owned by an older relative or friend or using existing health care professional contacts within social networks. Research was required to understand BP and its measurement. This was mainly online, for example searching for normal BP values and researching the health consequences of high BP. Although service users were advised to obtain a blood pressure reading from their local pharmacy most did not feel that this was a useful option for them. Many participants did not have access to a BP monitor and combined strategies to generate what they felt was an accurate measurement, increasing their confidence in the value they uploaded by triangulating between different methods of measurement. Their accounts of this (see table 2) illustrate the disjuncture between the expectations from the online service, which required the systolic and diastolic readings, and the experience of users, who often knew that their BP was normal but lacked access to the specific readings.

Table 2: Work required to generate a BP measurement though a combination of strategies

Case details	Measurement strategy	Account of the measurement process
Participant F	Remembered an old reading, knew that BP was usually normal and planned to get it checked before the next order	*"I think that was like from a doctor's that I had, like I think a few months beforehand. So, it was within normal range so it was fine and just remembered it …. so I knew that it wouldn't be like as reliable as doing a blood pressure straight away but within that, like I needed it quickly so I knew that normally like I'm fine with blood pressure so I just put in what I could remember from the last one, like it was normal …. So I think, if I was to like order again, I think I would maybe consider actually taking it like closer to the point"*
Participant O	Remembered that a previous reading had been normal and looked up a normal value online	*"Yeah, when the blood pressure thing came up I was a bit nervous … I knew that I had healthy blood pressure, so I could kind of estimate… for me I just sort of, googled what a healthy kind of reading was and vaguely remembered from my last time, anyway"*
Participant T	Estimated BP based on multiple previous readings from GP and home BP machine	*"So, the blood pressure I used an estimate of what the previous readings I'd had in the last twelve months. I'd had a GP visit and I had it checked. And then I'd had it checked a few times at home, 'cause we had a blood pressure monitor at home, that we were trying out and seeing if it worked or not. And all of those had been fine in the past. So I was fairly confident that it wouldn't have changed"*

As a result of the difficulty accessing BP measurements many participants found themselves negotiating their relationship with this new type of clinical service as they sought to understand whether this was a relationship of trust that required honest reporting of biometric measurements. They reflected on the whether their information would be reviewed by a clinician or a digital algorithm. They felt that communication with a clinician would require greater commitment to providing an accurate reading and they worked to

For peer review only

understand what might be a reasonable mix of measurement techniques to provide such a reading if they did not have access to the recent measurement requested by the service. They saw the need to provide BP as something that was required by the service and therefore a barrier to accessing contraception but also as something that might be important for their own health. Some users took on the new responsibility very seriously and took personal responsibility for providing an accurate measurement.

"I just suddenly had like a fear of god moment, where I was like, if I lie about this and then something does happen maybe they'll turn around and be like "well, you're not supposed to be over this weight or you're blood pressure has got to be this amount"…… things with your health, you've got to take seriously. It makes me nervous. So, even if it is the pill, which 9/10 isn't going to be an issue ….. Because I wasn't- there wasn't a face to it, I felt I really needed to make sure I was doing it right" Participant L

Others felt that this responsibility was shared with the online service and expected the clinicians/algorithms to identify inaccurate readings and uncover inappropriate strategies to collect them. For example, one participant who had guessed a reading that was biologically impossible and was contacted by a clinician from the service to find out when and how it was measured. She was then surprised and a little angry when her (untrue) explanation was accepted, feeling that the clinician should have been more questioning of the information she gave.

"The first message that came up was like "Oh you will be contacted by a nurse or something. To discuss.... further" …. I got a text message from the nurse saying how did you gather your blood pressure information. And I was like "Oh when I went to the clinic, she wrote it down for me". But I just entered it wrong…. And they said "Oh OK then, that's fine. Thank you. We'll let the nurse know…. I thought it was going to be much more nerve-wrecking, like calling my phone …". Participant G

This participant was prepared to compromise her health in order to obtain the contraception that she needed.

"Then I just had to google it, I just had to google "What is the correct blood pressure". And then one came up, they gave me a average. And I was like OK. Put that in…". Participant G

Participants wanted more information about BP and its importance in relation to the COCP and more help with obtaining a reading although none of the participants reported requesting help from the service. The digital interface seemed to communicate the BP measurement as the users' responsibility and the inputting of normal measurements as requirement. Participants felt that making contact might compromise their entitlement to the COCP and none did so unless the service contacted them first.

Throughout the interviews there was a marked difference between participants' description of measuring BP and their experience of measuring height and weight. They felt that monitoring their weight was a common health maintenance activity and they had been previously encouraged to do this. Height does not change in adulthood and most people

For peer review only

have had it measured at some time and remember this measurement at least approximately.

Discussion

The need to measure BP was experienced as an important barrier to access an otherwise convenient online contraceptive service. Significant work was required by users, the digital interface and clinicians to facilitate the process of BP measurement, communication and assessment. Users completed social, ethical and information gathering work to provide numerical BP values often combining strategies to maximise the validity of the reading uploaded, researching what is normal and negotiating what they felt was an appropriate commitment to providing accurate information. This negotiation referenced their need to access the pill urgently, feelings about a trusting relationship with the clinical service and concerns about the health implications of taking the pill with high BP. Better communication of the presence of the clinicians within the service and their willingness to support users would have made this process easier. Users found the measurement of height and weight straightforward and did not experience this as a barrier to access the service in any way.

Developing capacity to utilise effective, evidence based self-care for sexual and reproductive health is a public good[1] and may expand access to contraception[20], abortion[26] and testing for sexually transmitted infections[5]. However, self-care within health systems involves delegation and re-distribution of health work so that while it may allow people greater control over their lives it also brings new responsibilities[27] and new requirements for clinical support.

Whereas health policy discourses have tended to portray the digital interfaces that mediate digital health care as neutral channels with predictable outcomes[28], this study and others[15, 29] suggest that digital interfaces do important work in brokering (or not) relationships of trust within online clinical services and that these will influence user experience and clinical outcomes[28, 30]. New media for consultations inevitably change some aspects of clinical relationships[31] and at present very little is known about the 'rules of engagement' for the management of clinical consultations through online portals[16, 32]. Our study suggests that it is important that both user and provider are clearly present as individuals within the online communication process, that provider commitment to building a clinical relationship is clearly demonstrated and visible to the service user and that the health benefits of accurate reporting of biometric measurements are clear.

Self-monitoring blood pressure has become popular among those with long term conditions and GPs estimate that almost a third of patients with hypertension self-monitor their blood pressure[33]. Similarly, research among pregnant women with gestational hypertension shows that self-measurement with support and training is acceptable and empowering[34-35]. However, experience of self- measurement of blood pressure for the population of young contraceptive users in our study seems to be quite different as their need to self-measure is intermittent, they lack information and received no training on BP and its measurement.

Conclusions

Our findings support the view that digitally facilitated self-management will require close collaboration between users and providers, particularly in 'digital-first' services. The digital interface is a particularly important in communicating and supporting this collaboration and its importance is often underestimated. As digitally facilitated self-management becomes increasingly prevalent within health systems, we suggest that the digital interfaces that support them should:

1. Fully acknowledge the work that users, providers and digital interfaces do within online services.
2. Ensure that user and provider are clearly present as individuals within the online communication process, that provider commitment to building a clinical relationship is demonstrated and that the health benefits of the provision of accurate information are communicated.
3. Enable appropriate adaptation of clinical processes outside clinical contexts to facilitate self-care, for example asking whether BP was normal rather than asking for specific values.

The service evaluated is currently being re-designed in response to this research aiming to communicate better information about BP and its measurement, realistic strategies for BP measurement and a commitment to collaborative user/provider relationship.

Footnote 1: text message sent to users to remind them to check their blood pressure before they order their next supply of pills
You have 2 weeks supply left of your oral contraception. You can repeat order here: https://sh24.org.uk/coc_orders/new To successfully repeat you will need to know blood pressure. IF YOU DO NOT KNOW YOUR BLOOD PRESSURE you can find out how to check it here: https://sh24.org.uk/contraception/order#question_7 Text back if you would like help. Thanks, SH:24

Funding
This report is independent research funded by the National Institute for Health Research (Research for Patient Benefit Programme, PB-PG-0815-20009). The views expressed in this publication are those of the author(s) and not necessarily those of the NIHR or the Department for Health and Social Care

Competing interests
Paula Baraitser is clinical director of the online service studied and is one of the prescribers within this service. Since completion of this study, Hannah McCulloch has worked at the online service on a different research project. This work is part of a process of research on innovation at SH:24 where all innovation is the subject of research to ensure that learning is shared widely. SH:24 is a 'not for profit' organisation that provides health services to the National Health Service and sharing learning through innovation and research is one of the principles of the organisation.

Author statement
Paula Baraitser contributed to the conception and design of the study, oversaw data collection, contributed to data analysis and drafted the manuscript.

Hannah McCulloch contributed to data collection and analysis and contributed to the manuscript on the manuscript.
Alessandra Morelli contributed to data collection and analysis and commented on the manuscript
Caroline Free contributed to the conception and design of the study, contributed to data analysis and commented on the manuscript.

Data statement
The anonymised qualitative interviews are available from the authors on request.

Figure captions
Figure 1: The work done by the user, digital interface and clinician to support communication of accurate biometric measurements
References

1. Narasimhan M., Allotey P., Hardon A., Self-care interventions to advance health and wellbeing: a conceptual framework to inform normative guidance, British Medical Journal, 2019;365.

2. Armstrong. D. Actors, patients and agency: a recent history. Sociology of Illness and Health, 2014; 36: 163-174.

3. Lorig K., Sobel D., Stewart A., Brown B., Bandura A., Ritter P., Gonzalez V., Laurent D., Holman H. Evidence suggesting that a chronic disease self-management programme can improve health status while reducing hospitalization: a randomized controlled trial. Medical Care, 1999; 37: 5-14.

4. Mol, A. 'Living with Diabetes: Care Beyond Choice and Control.' The Lancet 2009; 373: 1756– 7.

5. Wilson W., Morris T., Syred J., Menon-Johansson A., Palmer M., Barnard S., Rezel E., Baraitser P. e-STI testing and results service: a single blind randomised controlled trial. Plos Medicine, 2017; 14: e1002479.

6. Eysenbach, G. What is e-health? Journal of Medical Internet Research, 2001; 3: e20.

7. Sheikh A., Sood H., Bates D. Leveraging health information technology to achieve the "triple aim" of healthcare reform. J Am Med Inform Assoc 2015; 4: 849-56.

8. World Health Organisation Guideline: Recommendations on digital interventions for health system strengthening, 2019 https://www.who.int/reproductivehealth/publications/digital-interventions-health-system-strengthening/en/

9. NHS digital. Personalised Health and Care 2020. National Information Board and Department of Health and Social Care, 2014. https://www.gov.uk/government/publicati§ons/personalised-health-and-care-2020 (Accessed 23/01/2020)

10. Lupton, Deborah, Self-Tracking Modes: Reflexive Self-Monitoring and Data Practices Paper for the ‘Imminent Citizenships: Personhood and Identity Politics in the Informatic Age’ workshop, 27 August 2014, ANU, Canberra http://dx.doi.org/10.2139/ssrn.2483549

11. Piras E. and Miele F.Clinical self-tracking and monitoring technologies: negotiations in the ICT-mediated patient–provider relationship, Health Sociology Review, 2017; 26: 38-53.

12. Greenhalgh, T., J. Wherton, P. Sugarhood, S. Hinder, R. Procter, and R. Stones. 2013. ‘ What Matters to Older People with Assisted Living Needs? A Phenomenological Analysis of the Use and Non-Use of Telehealth and Telecare.’ Social Science & Medicine 93: 86– 95.

13. May C., Finch T., Mair F., Mort M. Towards a wireless patient: chronic illness, scarce care and technological innovation in the United Kingdom. Social Science and Medicine, 2005; 61: 1485-94.

14. Mort, M., Finch T., May C. ‘ Making and Unmaking Telepatients: Identity and Governance in New Health Technologies.’ Science, Technology & Human Values,2009; 34: 9–33.

15. Mort, M., Roberts C., Callén B. ‘ Ageing with Telecare: Care or Coercion in Austerity?’ Sociology of Health & Illnes, 2013; 35: 799–812.

16. Rodgers M., Raine G., Thomas S. Informing NHS Policy in ‘digital-first’ primary care: a rapid evidence synthesis. Health Services and Delivery Research, 2019: 1-54 ISSN 2050-4357.

17. Baraitser P. and Cribb A. ‘Putting people in charge of their own health and care: Using meta-narrative review and the example of sexual health services to re-think relationships between e-health and agency. Health Expectations, 2019, 22; 5: 838-848.

18. Turner K., Looker K., Syred J., Zienkiewicz A., Baraitser P. Online testing for sexually transmitted infections: a whole systems approach to predicting value. PLoS One. 2019, 14(2): e0212420.

19. Hogenson, Eiluned & Jett-Goheen, Mary & Gaydos, Charlotte. (2019). An Analysis of User Survey Data for an Internet Program for Testing for Sexually Transmitted Infections, I Want the Kit (IWTK) in Maryland and Washington D.C. Sexually transmitted diseases, 2019; 46: 768-770.

20. Rezel-Potts. E., Palmer M., Free C., Baraitser P., The service users of online contraception: a cohort study. The Lancet, 2019, 394, S80.

For peer review only - http://bmjopen.bmj.com/site/about/guidelines.xhtml

21. FSRH, Faculty of Sexual and Reproductive Health Care UK Medical Eligibility Criteria for Contraceptive Use, 2016. https://www.fsrh.org/standards-and-guidance/uk-medical-eligibility-criteria-for-contraceptive-use-ukmec/ (accessed 27/11/2019)

22. World Health Organisation. Medical Eligibility Criteria for Contraceptive Use, 5th Edition, 2015. https://www.who.int/reproductivehealth/publications/family_planning/Ex-Summ-MEC-5/en/ (accessed 28/11/2019)

23. Oelkers WK. Effects of estrogens and progestogens on the renin-aldosterone system and blood pressure. Steroids. 1996;61(4):166–171.

24. Faculty of Sexual and Reproductive Health, Clinical Guidance on Combined Hormonal Methods of Contraception, 2019. https://www.fsrh.org/standards-and-guidance/documents/combined-hormonal-contraception/ (accessed 27/11/2019).

25. Lambeth, Southwark and Lewisham Public Health departments, Sexual and Reproductive Health Strategy 2019-2024, 2019, https://www.lambeth.gov.uk/sites/default/files/yh-lsl-sexual-health-strategy_0.pdf (accessed 20/11/2019)

26. Aiken A., Digol I., Trussel J., Gomperts R. Self reported outcomes and adverse events after medical abortion through online telemedicine population based study in the Republic of Ireland and Northern Ireland BMJ, 2017; 357:j2011

27. Lupton D. 'Quantified sex: a critical analysis of sexual and reproductive self-tracking using apps', *Culture, Health and Sexuality*, 2015; 17: 440 -453.

28. Lupton D. Towards sensory studies of digital health. Digital Health, 2017; 3: 1-6.

29. Pols J., Willems D., Innovation and Evaluation: taming and unleashing health care technology. Sociology of Illness and Health, 2011; 33: 484-498.

30. Fors V. and Pink S. Pedagogy as Possibility: Health Interventions as Digital Openess. Social Sciences, 2017, 6: 59-71.

31. Atherton H and Ziebland S. What do we need to consider when planning, implementing and researching the use of alternatives to face-to-face consultations in primary healthcare? Digital Health, 2016; 2:1-13.

32. Atherton H., Pappas Y., Heneghan C., Murray E. Experiences of using email for general practice consultations: a qualitative study. British Journal of General Practice, 2013; 63: e760-767.

33. McManus RJ, Wood S, Bray EP, et al. Self-monitoring in hypertension: a web-based survey of primary care physicians. Journal of Human Hypertension. 2014; 28: 123–127.

For peer review only

34. Hinton L, Tucker K, Greenfield S, Hodgkinson J, Mackillop L, McCourt C, Carver T, Crawford C, Glogowska M, Locock L, Selwood M, Taylor KS, McManus RJ. Blood pressure self-monitoring in pregnancy (BuMP) feasibility study; a qualitative analysis of women's experiences of self-monitoring. BMC Pregnancy Childbirth, 2017; 19: 17-42.

35. Tucker K., Taylor K., Crawford C., Hodgkinson J., Bankhead C., Carver T., Ewers E., Glogowska M., Greenfield S., Ingram L., Hinton L., Khan K., Locock L., Mackillop L., McCourt C., Pirie A., Stevens R., McManus R. Blood pressure self-monitoring in pregnancy: Examining feasibility in a prospective cohort study. BMC Pregnancy and Childbirth, 2017; 17: 442-452.

For peer review only

User

Understands requirement for BP, height and weight.

Accesses information on BP height and weight as required.

Accesses a BP and BMI measurement.

Assesses whether the measurement is normal.

Uploads the readings when requested within the electronic order journey.

Communicates with the clinician as necessary.

Digital interface

Requests information on BP, height and weight.

Presents information on BP and BMI including normal values and the risks of taking the COCP with high BP or BMI.

Stores the BP or BMI on the clinical record and highlights it when outside the normal range to draw the attention of the clinician

Enables communication between service user and provider through text message or telephone.

Records the communication for reference.

Clinician

Completes a clinical assessment of any readings that are highlighted within the system.

Communicates with the service user if the readings are outside the normal range.

Assesses the validity of the readings based on the communication with the user.

Requests additional measurements if appropriate.

Decides whether to prescribe the COCP or not.

Effectively communicates this decision

Appendix A – Information provided by the service on the risks of taking the COCP with high blood pressure

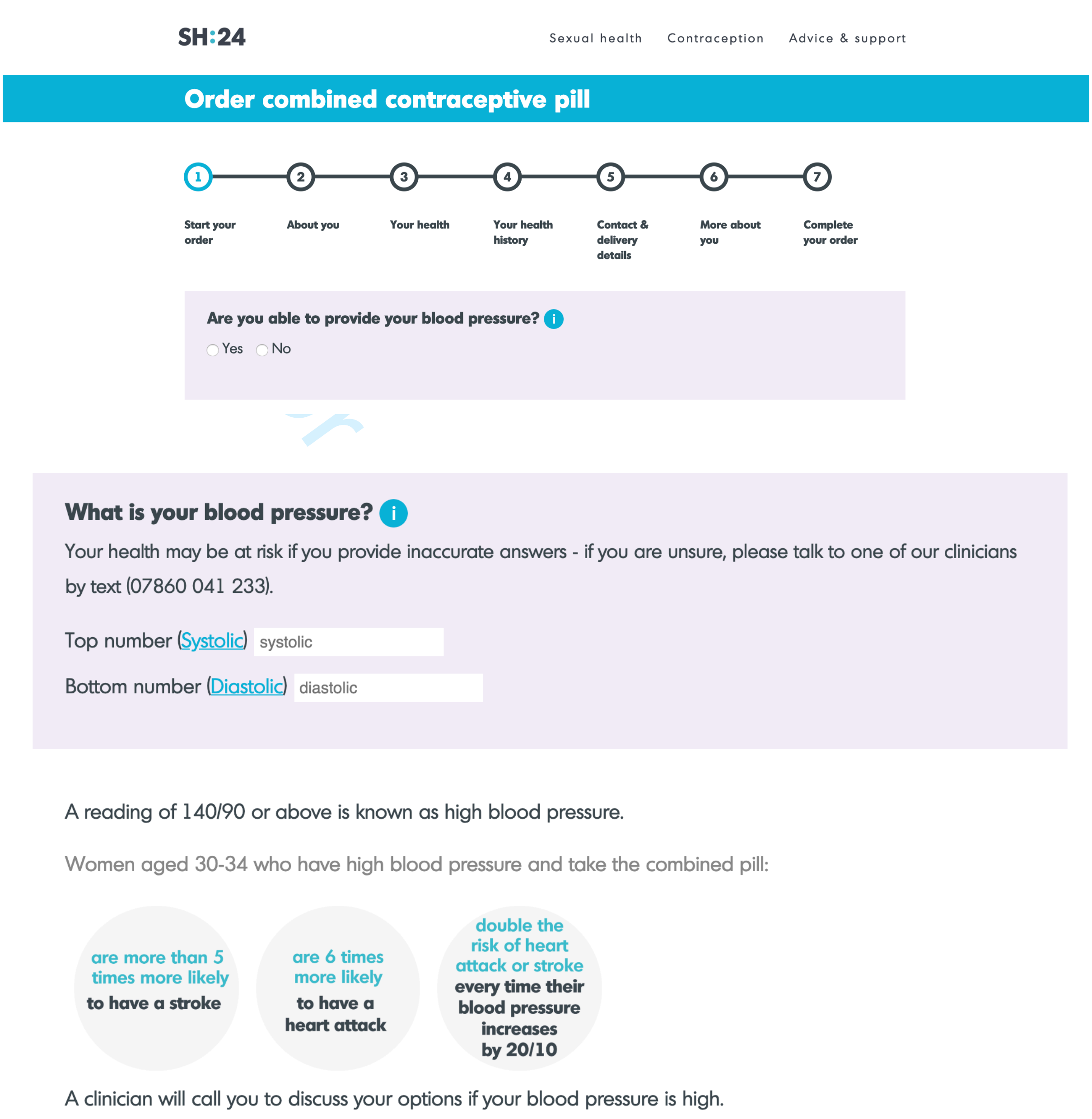

Appendix B: Qualitative paper coding tree

Theme	Sub-themes	Notes
The practical realities of obtaining contraception from traditional services and the choice to order the pill online	An alternative to inconvenient existing options<br><br>Fits into my lifestyle<br><br>A solution to an urgent situation	Navigating access to contraception with time pressure - Time and urgency is of fundamental importance in this assessment. Reference negative case study of person who did not have time pressure
A new contraceptive service option generates new work, new roles and new responsibilities.	Social work (Drawing on existing relationships to access a BP reading)<br><br>Ethical work (Negotiating an ethical position under pressure to obtain contraception)<br><br>Research work (online research to find out what is normal and the consequences of taking the pill with high BP).	By changing the frame of access and the nature of the interface with the health system we have created a new option with a new set of practical realities.<br><br>No existing pathways, so created new pathways, used chance events. Translating past experience into this new context.<br><br>Differences between strategies to measure BP (unfamiliar) and height/weight (familiar)
Ideas about blood pressure and its measurement.	Blood pressure as an intangible concept<br><br>Blood pressure as linked to own health and normality – from past clinical experiences<br><br>BP as a medical thing whereas BMI is more in the public domain.<br><br>Blood pressure and how recent it needs to be?	Consequences of these ideas for choice of measurement strategy.
Responsibility and risk	Negotiating a clinical relationship with a faceless service<br><br>The characteristics of the digital interface that supported or did not support effective communication.	

For peer review only
